# Seizure and Status Epilepticus in Human Organophosphate Poisoning: A Narrative Review

**DOI:** 10.3390/neurolint18040065

**Published:** 2026-03-30

**Authors:** Giuseppe Magro, Oreste Marsico, Federico Tosto, Concetta Lobianco, Laura Rapisarda, Giovanni Mastroianni, Angelo Pascarella

**Affiliations:** 1 Neurology Department, Lamezia Terme Hospital, 88046 Catanzaro, Italy; 2 Unit of Neurology, Sant’Elia Hospital, 93100 Caltanissetta, Italy; 3 Department of Medical and Surgical Sciences, Magna Graecia University, 88100 Catanzaro, Italy

**Keywords:** organophosphate, nerve agents, acetylcholinesterase inhibition, seizures, status epilepticus, nonconvulsive status epilepticus, benzodiazepines, benzodiazepine resistance, receptor trafficking

## Abstract

Organophosphate (OP) exposure can trigger seizures within minutes and can rapidly evolve into status epilepticus (SE). Early seizure generation is plausibly driven by acetylcholinesterase inhibition, leading to central cholinergic overstimulation. With increasing seizure duration, experimental data are consistent with a time-dependent shift toward glutamatergic maintenance (NMDA/AMPA), oxidative stress, neuroinflammation, and progressive failure of GABAergic inhibition. This framework predicts a narrow window in which benzodiazepine (BDZ) monotherapy is most effective and a rising probability of BDZ non-response when seizures are prolonged, while anti-glutamatergic strategies may retain relative efficacy later in the course. This narrative review integrates clinical phenomenology, diagnostic limitations, and mechanistic evidence to propose an operational approach for OP-related seizures and SE in emergency settings. We discuss a pragmatic “Stage 1 Plus” framing for patients presenting after prolonged seizures or in non-convulsive SE with coma. Human evidence remains limited and heterogeneous, and inference is constrained by confounding due to delayed recognition, variable decontamination/resuscitation pathways, sparse EEG confirmation, and selection bias in mass-casualty reporting.

## 1. Introduction

**Figure 1:**
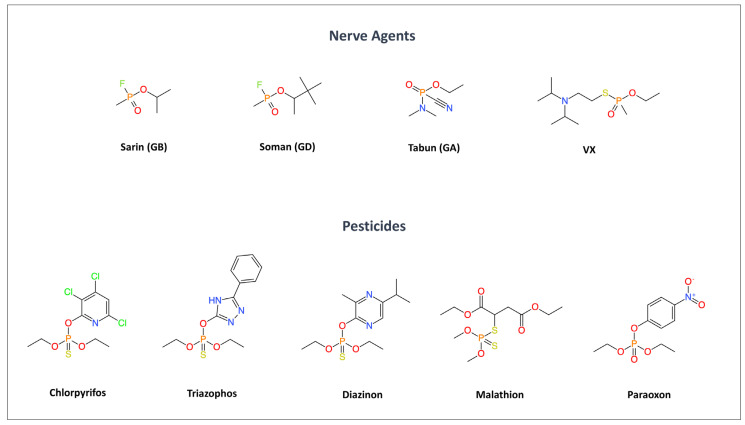
Chemical structures of main nerve agents and organophosphate pesticides. The figure shows the chemical structures of selected nerve agents and widely used organophosphate pesticides. All compounds share an organophosphate core responsible for acetylcholinesterase inhibition, while structural variations in substituents account for differences in lipophilicity, environmental persistence, and toxicity (Created with BioRender.com).

Organophosphates (OPs) are among the most potent neurotoxic substances known; they are a chemically diverse group of substances widely used in agriculture and industry, and developed as chemical warfare nerve agents. OPs are esters, amides, or thiol derivatives of phosphoric or phosphonic acid, characterized by a central phosphorus atom bonded to oxygen or sulfur and various substituents [1]. The structural variety of different alkoxy or amino groups influences their physicochemical properties, lipophilicity, and toxicity. Based on their applications, OPs are generally classified into potent nerve agents developed for military purposes (e.g., sarin, soman, tabun, and VX) and agricultural pesticides such as chlorpyrifos, malathion, parathion, diazinon, and paraoxon [1,2] (see Figure 1).

They have the property of inducing rapid and severe dysfunction of the central nervous system through the irreversible inhibition of acetylcholinesterase. The accumulation of acetylcholine leads to a cholinergic crisis that affects the peripheral, autonomic, and central nervous systems [3]. Severe OP poisoning presents with numerous acute clinical symptoms, including muscle weakness, respiratory distress, excessive sweating and mucosal secretions, diarrhea, decreased consciousness, epileptic seizures, and coma. Despite their clinical importance, seizures and status epilepticus (SE) in OP poisoning are often unrecognized and undertreated, especially in mass-casualty or resource-limited environments. OP poisoning is a medical emergency that frequently requires immediate, multidisciplinary treatment and combination therapies, including a muscarinic antagonist (such as atropine), an acetylcholinesterase reactivator (oximes), and benzodiazepines (BDZ) for seizure control [4,5,6,7]. OP poisoning remains a significant global public health issue, causing more than 200,000 deaths each year, involving intentional self-poisoning, occupational exposures, and mass exposure events [8]. The burden is especially high in areas where highly toxic compounds are readily accessible and prompt medical care and antidotes are not readily available. OP poisoning represents one of the most common methods of suicide in rural agricultural communities. Occupational and accidental exposures further contribute to the burden, especially among agricultural workers [8].

While hemodynamic instability and respiratory failure have traditionally been considered the leading causes of death, seizures and SE are critical factors contributing to mortality and long-term neurological damage [6]. In this review, we aim to provide a thorough overview of the pathophysiology, clinical features, and management of OP-induced neurotoxicity, with a particular focus on seizures and SE. We also discuss the emerging concept of SE early pharmacoresistance and its potential implications for treatment strategies in emergency settings.

## 2. Clinical Features and Differential Diagnosis of Organophosphate Poisoning

OP poisoning is a clinical emergency characterized by a wide spectrum of clinical manifestations resulting from inhibition of the acetylcholinesterase enzyme [3,9]. This enzymatic blockade causes an excessive increase in acetylcholine at synaptic junctions and overstimulation of muscarinic and nicotinic receptors [10].

The clinical presentation is complex and depends on the dose and duration of exposure. The hallmark of OP intoxication is acute cholinergic syndrome, consisting of a multisystemic involvement. Symptoms related to nicotinic effects include muscle fasciculations, weakness, paralysis, tachycardia, and arterial hypertension. Muscarinic manifestations include miosis, sialorrhea, lacrimation, bronchial secretions, bronchospasm, bradycardia, abdominal cramping, vomiting, and diarrhea [3,11]. Table 1 summarizes the clinical features of OP intoxication.

Central nervous system involvement ranges from confusion to epileptic seizures, central respiratory depression, and coma [9,12]. Acute respiratory failure due to a combination of bronchial hypersecretion, bronchoconstriction, and neuromuscular weakness is the primary cause of mortality [3]. Patients who survive the acute phase may develop the so-called intermediate syndrome, usually occurring 1–4 days after OP exposure, characterized by proximal limb muscle weakness, single or multiple cranial nerve palsies, and respiratory muscle involvement [11]. Distal sensorimotor neuropathy is less common and can emerge weeks later [10].

OP poisoning is challenging to diagnose and must be distinguished from other causes of cholinergic intoxication. For instance, carbamate poisoning has similar clinical features with a shorter duration due to reversible enzyme inhibition [3,13]. Other conditions include toxicity from nicotinic agents, mushroom poisoning, opioid overdose, and brainstem lesions, which may mimic respiratory compromise and altered mental status. Moreover, severe asthma, gastroenteritis, or myasthenic crisis may resemble isolated components of OP toxicity, lacking multi-system involvement [11,12]. Diagnosis relies on an accurate medical history, clinical recognition of symptoms, and serological measurement of cholinesterase activity [13]. However, serological measurements should not delay treatment, as early identification is crucial and prompt treatment significantly improves outcomes, reduces complications, and lowers mortality.

## 3. Mechanism of Organophosphate-Induced Seizure and Status Epilepticus

Nerve agents can induce neurotoxic effects by irreversibly inhibiting acetylcholinesterase, leading to sustained accumulation of acetylcholine at the synapse. Overstimulation of central muscarinic receptors is the underlying mechanism of seizures and can rapidly progress to SE [9,14,15]. In this phase, seizure activity depends on the perpetuation of cholinergic overstimulation; over time, excessive neuronal firing leads to involvement of other neurotransmitter systems [15], characterized by a progressive increase in synaptic glutamate, NMDA receptor overactivation, increased calcium influx, and progressive failure of GABAergic inhibition, which together promote seizure activity [15]. Increased NMDA receptor activation impairs intracellular calcium homeostasis, triggering activation of calcium-dependent proteases, mitochondrial dysfunction, and phosphorylation-dependent modification of ion channels and receptor subunits [15]. This hyperexcitability leads to the activation of NADPH oxidase isoforms and the excessive production of reactive oxygen species, lipid peroxidation, DNA damage, and redox-sensitive modulation of glutamate receptors and voltage-gated ion channels, thereby further amplifying excitotoxic signaling. This process is mirrored by a neuroinflammatory response, including microglial activation, astrocytic reactivity, and upregulation of pro-inflammatory cytokines such as interleukin-1β and tumor necrosis factor-α, which lower the seizure threshold and promote long-term synaptic reorganization [10,14]. As SE persists, excitotoxic and inflammatory mechanisms induce injury in limbic and paralimbic regions, particularly in the amygdala, piriform cortex, hippocampus, and entorhinal cortex [9,14]. Experimental models using soman, sarin, diisopropyl fluorophosphate, and related OP demonstrate that the severity and duration of SE directly correlate with the extent of damage and long-term network remodelling within the hippocampal–limbic network [16,17]. The pathological changes related to neuroinflammation, oxidative stress, and maladaptive synaptic plasticity promote spontaneous recurrence of seizures and stabilize epileptogenic networks closely resembling acquired temporal lobe epilepsy [10,14,17].

Of note, the use of atropine, oximes, and BDZ reduces acute mortality and suppresses early seizures, but fails to prevent epileptogenesis [5,9,15]. The shift from primarily cholinergic to glutamatergic and inflammatory response may reflect the emergence of pharmacoresistant epilepsy following OP SE [10,14,16].

### Lessons from Experimental Models and Clinical Data

Experimental models have shown that OPs and nerve agent exposure quickly induce generalized convulsive seizures that often develop into self-sustaining SE, marked by early pharmacoresistance to BDZ and increasing excitotoxic injury [18]. Seminal works demonstrated that cholinergic-induced SE is linked to activity-dependent trafficking and internalization of synaptic GABA-A receptors, along with upregulation of NMDA and AMPA glutamate receptors, thereby creating a state of maladaptive hyperexcitability [19]. From a therapeutic standpoint, these trafficking-driven shifts imply that the window for BDZ monotherapy closes quickly (within 10 min) [6,20]. In contrast, NMDA antagonism remains comparatively effective as the seizure network becomes glutamate-dependent. In cholinergic/OP models, outcomes improve when treatment is initiated early with mechanism-based polytherapy that restores inhibition while dampening NMDA-mediated excitation (e.g., BDZ plus ketamine), rather than relying on sequential stepwise therapy after prolonged seizures [21,22,23]. This experimental trajectory mirrors the clinical challenge in OP-related SE, where peripheral antidotal therapy may only stabilize systemic cholinergic toxicity. Yet, seizures persist and require polytherapy antiseizure management to limit excitotoxic injury and longer-term sequelae [24].

OP-induced SE has distinct features and similarities compared with other models of self-sustaining SE. In a direct comparative study, pilocarpine, diisopropylfluorophosphate (DFP), and soman differed in seizure onset, BDZ sensitivity, and neuronal injury profile [25]. DFP and soman progressed more rapidly to persistent BDZ-resistant seizure activity, whereas pilocarpine-induced SE remained comparatively more diazepam-responsive in the same model [25]. The key difference lies in seizure initiation: OP intoxication begins with irreversible acetylcholinesterase inhibition and a systemic cholinergic toxidrome, whereas pilocarpine and kainate induce SE through different initiating mechanisms [26]. Once SE is established, however, downstream biology converges in part. Evidence from self-sustaining SE models, supported by clinical observations in human SE, links longer seizure duration to progressive loss of GABA-mediated synaptic inhibition and a consequent decline in BDZ responsiveness [23,25].

Neurosteroids constitute an additional point of convergence shared across these models. Reddy and collaborators demonstrated that pregnane neurosteroids, including allopregnanolone and ganaxolone, suppress SE in DFP, soman, and pilocarpine models alike by enhancing tonic inhibition at extrasynaptic GABA-A receptors, which remain functionally intact even as synaptic receptors are internalized during prolonged seizures [27]. Neuroactive steroids also protect against pilocarpine- and kainic acid-induced seizures and SE [28]. Allopregnanolone and related neurosteroids are markedly reduced in the cerebrospinal fluid of patients with SE [29]. These findings support neurosteroid signalling as a shared therapeutic axis across SE triggers.

Regarding EEG spectral dynamics, the available OP data do not identify a kainic acid-like theta surge as the dominant signal. In the kainic acid model, theta power rises to about 150% of baseline 3 h after kainic acid injection and then returns toward baseline as convulsive seizures fully emerge; in that model, earlier onset of the first convulsive seizure with loss of posture predicts earlier spontaneous recurrent seizures [30]. In the DFP model, the reported signal is different: the theta-delta ratio falls acutely, and broadband power rises during SE. A higher acute theta-delta ratio correlates with higher spike rates at 3 and 7 days, whereas greater broadband power during SE and slower broadband recovery correlate with greater spontaneous recurrent seizure burden [31]. A transient theta rise analogous to that described in the kainic acid model has not yet been reported in the available OP data, favoring a possible distinctive pattern.

**Figure 2:**
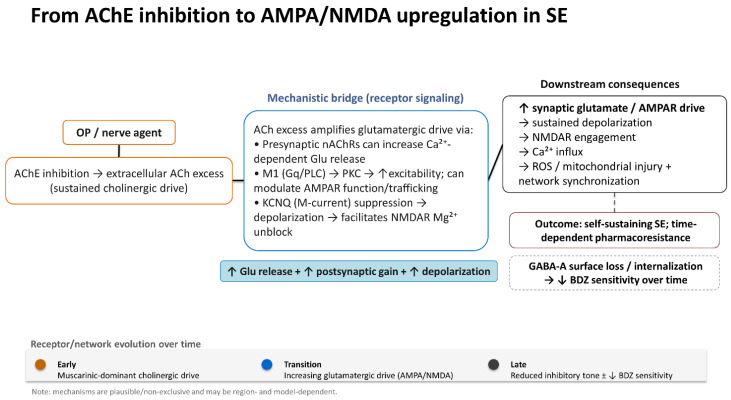
Organophosphates/nerve agents inhibit acetylcholinesterase, leading to extracellular acetylcholine accumulation and an early hypercholinergic seizure-driving phase [15]. A commonly used framework proposes a transition from early cholinergic initiation to seizure maintenance, increasingly supported by glutamatergic mechanisms [15,26]. Acetylcholine excess can amplify excitatory drive through M1-dependent suppression of KCNQ2/3 (M-current) and enhanced glutamatergic transmission [26]; in selected circuits, presynaptic α4β2 nicotinic receptors can further facilitate Ca^2+^-dependent glutamate release [32]. M1 signalling can also engage kinase pathways that modulate AMPAR GluA1 phosphorylation and surface insertion, increasing postsynaptic gain [33]. The resulting depolarization relieves the voltage-dependent Mg^2+^ block of NMDARs, enabling Ca^2+^ influx and downstream excitotoxic/oxidative injury pathways [34,35]. In parallel, prolonged seizures are associated with erosion of synaptic GABA-A receptor-mediated inhibition and time-dependent reduction in benzodiazepine responsiveness [20,23]. Abbreviations: ACh acetylcholine; AChE acetylcholinesterase; AMPAR AMPA receptor; BDZ benzodiazepines; Ca^2+^ calcium; DAG diacylglycerol; GABA-A gamma-aminobutyric acid type A receptor; Gq/11 Gαq/11; IP3 inositol-1,4,5-trisphosphate; KCNQ2/3 Kv7.2/7.3 channels (M-current); M1 muscarinic M1 receptor; Mg^2+^ magnesium; mAChR muscarinic acetylcholine receptor; nAChR nicotinic acetylcholine receptor; NMDAR NMDA receptor; OP organophosphate; PLCβ phospholipase C beta; PKC protein kinase C; ROS reactive oxygen species; SE status epilepticus.

Figure 2 summarizes the proposed mechanism for OP-induced seizures and SE.

A recent critical review introduced the clinical concept of Stage 1 Plus, defined as a SE that is potentially refractory to first-line BDZ therapy and requires polytherapy at first evaluation. The Stage 1 Plus definition includes any of the following: prolonged seizure activity (>10–30 min depending on semiology), acute symptomatic etiologies (especially central nervous system (CNS) etiologies), and non-convulsive SE (NCSE) with coma [36,37,38,39]. These considerations stem not only from experimental models, but also from human studies exploring resistance to BDZ in SE. Among these, the seminal work by Burman et al., comparing data from low-income and high-income countries, highlights that longer delays to first-line BDZ treatment (more common in resource-limited settings) are associated with a higher proportion of BDZ-refractory SE, consistent with a time-dependent emergence of pharmacoresistance as seizure duration prolongs [20]. Longer delays to the first BDZ are significantly associated with later SE termination [40]. Moreover, the study by Llauradó Arnau et al. showed that SE triggered by acute brain injury often responds poorly, even when BDZs are administered promptly, supporting early combined therapy for these etiologies [41], thereby reinforcing the Stage 1 Plus concept. In a recent letter in Epilepsia, Wasterlain et al. explicitly propose that nerve agent-induced SE be categorized as Stage 1 Plus, given its severity, high mortality, and early treatment failure [42]. Moreover, in terrorist attacks, treatment of OP exposure is often delayed, especially in the civilian sector, where early treatment is unlikely [6].

## 4. Lessons from the Battlefield

Large-scale nerve-agent releases during war or terrorism provide rare “human-scale” evidence on the tempo and severity of central toxicity from OP acetylcholinesterase inhibitors. Yet, electroclinical confirmation is seldom feasible (no EEG, competing airway priorities, and difficult, timely response). Consequently, seizures are often reported only as “convulsions” and SE is likely under-recognized, particularly early after exposure when hypoxemia, secretions, and triage dominate management [43,44].

The Matsumoto (1994) and Tokyo subway (1995) sarin attacks illustrate key operational constraints of urban toxicologic mass-casualty events: overwhelming patient volumes, prioritization of airway/ventilatory support, and documented secondary exposure of healthcare personnel, underscoring the importance of early decontamination, staff protection, and immediate access to antidotes and BDZ [45,46,47]. Military reports of tabun and sarin exposure during the Iran–Iraq War (1980–1988) describe seizures and coma in severely exposed casualties, consistent with rapid CNS involvement; however, systematic electroclinical classification is not feasible in battlefield conditions [2,48,49].

In the Ghouta (Damascus) sarin incident (2013), a nerve-agent toxidrome with seizures in 24 of 130 victims (18.5%) and loss of consciousness in 53 of 130 (40.8%) was identified [43]. The findings support a substantial burden of severe seizure activity, although the authenticity and selection bias of studies cannot be fully controlled. Furthermore, it is interesting to note that the data were derived mainly from videos and reports, and no BDZ or oxime was administered during the Syrian attack [43].

Across battlefield and civilian mass exposure scenarios, seizures were repeatedly documented as a prominent manifestation of OP toxicity. Gunderson and colleagues have already emphasized that seizures are dose-dependent and centrally mediated, with rapid blood–brain barrier penetration by nerve agents [48]. In several historical series, seizures occurred within minutes of exposure and were followed by prolonged coma, a clinical trajectory highly suggestive of SE. Although EEG confirmation was unavailable, the phenomenology closely mirrors experimental OP-induced SE. Long-term follow-up of sarin-exposed individuals in Japan revealed persistent EEG abnormalities and epilepsy, supporting the concept that acute OP-induced seizures may initiate epileptogenesis when inadequately controlled [50].

### 4.1. Lessons from the Emergency Rooms and Domestic Incidents

Complementing the battlefield narrative, non-warfare domestic exposures, household accidents, community outbreaks, and routine emergency department (ED) admissions provide higher-resolution clinical data and still show that seizures can be a prominent early CNS manifestation of OP toxicity. In the 2020 Eluru (India) outbreak, seizures were reported in 84% of cases, and toxicologic testing supported OP exposure with triazophos detected in blood and metabolites in urine, reinforcing how abruptly a “seizure-first” presentation can present even in community-scale events [51]. In a large ED cohort from La Paz (Bolivia), including acute OP/carbamate intoxications, seizures were documented in ~7%, showing that, even in standard ED workflows (non-mass-casualty), seizure recognition is clinically relevant and not rare [52]. Hospital cohorts focused on specific OPs show comparable data: in chlorpyrifos and pesticide poisoning, seizures occurred in ~7.5%, framing seizures as part of the severe toxidrome spectrum rather than an exceptional complication [53]. Similarly, in a cohort of chlorpyrifos intoxication, seizures were explicitly listed among severe complications (alongside respiratory failure and acute kidney injury) [54]. In elderly-focused OP cohorts, seizures may be less frequent (e.g., 4.2%) yet remain present and clinically meaningful in a population with a high burden of comorbidity [55]. Beyond acute symptomatic seizures, a nationwide retrospective cohort analysis found OP poisoning associated with a substantially higher subsequent seizure risk (adjusted HR 3.57), supporting the concept that OP exposure can mark (or unmask) a longer seizure-vulnerability trajectory beyond the acute cholinergic crisis [56].

### 4.2. Exposure Route

In OP poisoning, both the route of exposure and the specific agent are important. Outside of warfare, the most severe civilian cases usually result from deliberate oral ingestion of agricultural pesticides, while unintentional poisoning is less common and typically occurs due to occupational or domestic exposure [3,4]. Nerve-agent exposure differs again: inhaled vapor acts within seconds to minutes, whereas liquid dermal exposure may be delayed for hours [7]. Human data do not support a uniform OP toxidrome. In prospective cohort studies, dimethoate, chlorpyrifos, and fenthion showed significant differences in clinical course and outcomes [57]. Dimethoate was associated with marked hypotension that progressed to distributive shock, whereas fenthion often exhibited fewer early cholinergic signs and a later decline [57,58]. Profenofos and prothiofos also displayed relatively mild initial cholinergic signs despite severe acetylcholinesterase inhibition, followed by delayed respiratory failure and death [59]. Direct human studies comparing individual OP insecticides and distinct seizure patterns remain limited. In human poisoning, the specific agent seems to modify the timing and prominence of central neurotoxicity more clearly than seizure semiology [57,59]. A recent profenofos case, presenting with SE 36 h after ingestion and no peripheral cholinergic signs, is consistent with delayed, mainly central neurotoxicity in some lipophilic compounds [60].

## 5. Treatment

Vapor and systemic exposure to OP can lead to rapidly evolving and potentially lethal manifestations requiring immediate evaluation and treatment. All personnel involved with the care of nerve agent victims need to wear personal protective equipment until the patient is decontaminated. Victims of OP intoxications are decontaminated with extensive showers; if the exposure level is minimal and the patient has only minor symptoms (such as miosis or secretions), running water over the eyes only is required to minimize eye injury [7,9]. The mainstay of treatment for OP intoxication is based on timely administration of atropine (a muscarinic antagonist), oximes (mainly pralidoxime), and benzodiazepines [7,9].

There are no robust dose–response data from studies examining the optimal dosing of atropine in OP poisoning. As a result, many recommendations on atropine dosages and the timing of administration have been made in recent years (ranging from 23.4 mg over 8–1380 min to 75 mg over 25–4440 min) [61].

Atropine should be administered as soon as possible, as atropinization reduces time-to-death and thereby lowers mortality [62]. Oximes are acetylcholinesterase-reactivating agents, effective only on non-aged enzymes; therefore, administration should be performed within 48 h of poisoning [9].

Regarding oximes, a recent meta-analysis on OP poisoning, comparing patients receiving atropine alone or atropine plus pralidoxime, showed a significantly higher risk of mortality (*p* = 0.020) and a longer length of stay (*p* < 0.001) in the atropine plus pralidoxime patient group. This evidence calls into question the routine application of pralidoxime in OP poisoning [63].

The available studies are heterogeneous and of low quality regarding clinical management of OP poisoning: there are few high-quality randomized clinical trials, and many are observational studies with small sample sizes. This limits the strength of therapeutic recommendations. In the USA, in 2002 and 2006, to optimize OP poisoning management, the FDA approved two autoinjectors: (1) a dual-chamber autoinjector containing 2.1 mg of atropine and 600 mg pralidoxime, named “DuoDote”, for civil use; (2) an autoinjector containing separated atropine and pralidoxime, named ATNAA, mainly used in military settings [9]. DuoDote in the USA is administered by the Centers for Disease Control and Prevention, which can supply nerve agent antidote packages to 90% of the population within 1 h [7,9].

SE management in nerve agent poisoning, then, is particularly difficult, since it does not respond to common antiseizure medications (ASMs) whose mode of action relies upon tamping down spread from one brain location to another [7]. BDZ, mainly diazepam, are effective for this form of seizure only within a very narrow time window, and treatment is often delayed, possibly resulting in an already BDZ-resistant state. BDZ are usually effective when given early after OP nerve agent exposure. Delayed administration may instead increase the risk of seizure recurrence. In general, then, the possibility of an OP nerve agent exposure requires an organized and coordinated plan involving local hazmat teams, police, experts, as well as a network of medical centers where well-trained and well-equipped staff are prepared to treat the expected large number of victims [9].

## 6. Long-Term Outcomes: Cognitive Impairment, Psychiatric Disease, and Epilepsy Risk

Long-term neurological and neuropsychiatric sequelae following OP exposure represent a major burden among both survivors of acute intoxication and chronically exposed populations [9]. Epilepsy, cognitive impairment, and psychiatric disorders represent the main chronic neurological diseases [9,10,64,65]. Converging experimental and clinical data describe a persistent pathophysiological cascade involving excitotoxicity, oxidative stress, neuroinflammation, and maladaptive network reorganization extending beyond the acute phase [10,66]. Moreover, SE after acute OP poisoning is strongly associated with persistent structural and functional brain alterations [9], and emerging evidence suggests that even low-dose, sub-chronic exposure can lead to significant neurobiological alterations [67].

Cognitive dysfunction is among the most consistently reported long-term consequences. Survivors of acute pesticide poisoning frequently develop persistent cognitive decline accompanied by structural brain abnormalities, mainly basal ganglia lesions [68]. Preclinical studies demonstrate that even low-dose OP exposure disrupts medial prefrontal cortex–dependent executive function and synaptic integrity [67]. In agricultural communities, cumulative exposure to OPs has been associated with reduced neurobehavioral performance, particularly in domains involving attention, memory, and executive function [69]. Vulnerability is particularly pronounced during development. Prenatal and childhood exposure to OPs is linked to altered functional brain connectivity, deficits in working memory, executive dysfunction, and reduced prefrontal cortex activation [70,71]. School-age children with measurable pesticide burdens show lower IQ indices, impaired reasoning and memory performance, and poorer performance in the attention and processing speed domains [72,73]. Chronic exposure to OPs is a recognized risk factor for neurodegenerative diseases, including Alzheimer’s and Parkinson’s disease [74]. Increased markers of cellular senescence (p16 and p21) have been demonstrated in preclinical models, suggesting OPs accelerate brain aging [75]. Neuroinflammation has been proposed as a key pathogenic driver [76], with studies linking diazinon and malathion to hippocampal-dependent memory deficits, amyloid-β accumulation, and disruption of the microbiota–gut–brain axis [74,77,78,79].

Psychiatric outcomes, including depression, anxiety, post-traumatic stress disorder (PTSD), and affective disturbances, are frequently reported in survivors of OP poisoning [9,64,65]. While the psychological trauma of a life-threatening event contributes to these symptoms in survivors of nerve agent exposure, preclinical evidence points to a biological basis for these behaviors. Animal models have shown that anxiety-like behaviors correlate with specific neuropathology in the basolateral amygdala, suggesting that OP-induced neurotoxicity directly disrupts the neural circuitry regulating emotional processing [9,79]. Cumulative OP exposure in agricultural settings correlates with reduced neurobehavioral performance and executive dysfunction, particularly when biomarkers confirm cholinesterase inhibition [69,80].

Epileptogenesis represents a critical long-term complication. The acute OP poisoning SE may contribute to the epileptogenic processes. Persistent oxidative stress and alterations in network connectivity correlate with seizure burden [81]. Experimental models demonstrate the association between hippocampal neuronal loss, mossy fiber sprouting, blood–brain barrier dysfunction, and subsequent emergence of seizures, consistent with acquired epilepsy [17,31,66]. In rat models, early electrophysiological abnormalities and changes in neural oscillatory activity, such as alterations in the theta-delta ratio, high-frequency oscillations, following acute intoxication, may predict later chronic epilepsy development [31,82]. Chronic neuroinflammation remains central, triggering sustained glial activation and oxidative stress [76,78]. Crucially, blood–brain barrier leakage allows peripheral inflammatory cells and toxins to enter the brain, further increasing neuroinflammation and epileptogenesis [66]. Targeting these pathways might represent a promising therapeutic frontier [10,67].

Ultimately, considering the significant long-term risks for cognitive decline, mood disorders, and epilepsy, longitudinal neurological and psychiatric follow-up must be integrated into the clinical management of high-risk individuals [64,66,68,75].

## 7. Conclusions

This narrative review aimed to study the association between OP exposure and epileptic seizures, which can evolve into self-sustaining SE. Early seizure generation is cholinergic-driven, but persistence appears attributable to glutamatergic drive, oxidative stress, neuroinflammation, and progressive failure of GABAergic inhibition, all of which together are consistent with a rapidly shrinking window for BDZ monotherapy efficacy in SE. Therefore, in severe OP/nerve-agent poisoning, seizure control should be managed as a parallel life-threatening target rather than a secondary complication of the peripheral toxidrome. When a seizure is going on for more than 10–30 min, and NCSE with coma is clinically plausible, OP-related SE can be operationally framed as a Stage 1 Plus phenotype. This framework is probably BDZ-refractory at first evaluation and therefore represents a candidate for early and mechanism-based polytherapy, including BDZ plus a complementary ASM targeting glutamatergic-driven maintenance. Our study highlights that human evidence remains limited and heterogeneous, and key confounders include delayed recognition, limited EEG confirmation, and selection bias in mass-casualty reports. Thus, more studies are needed to investigate clinical features and optimal care for OP-related seizures to avoid long-term consequences, including epilepsy and cognitive disorders.

## Figures and Tables

**Table 1:** Clinical features of organophosphorus compound poisoning.

Autonomic Parasympathetic Symptoms (Muscarinic Receptors Overstimulation)
Sialorrhea
Vomiting
Diarrhoea
Hypotension
Bradycardia
Miosis
Lacrimation
Urination
Bronchospasm
Bronchorrhoea
Autonomic sympathetic symptoms (nicotinic receptors overstimulation)
Hypertension
Tachycardia
Sweating
Mydriasis
Overstimulation of nicotinic and muscarinic receptors in the CNS
Confusion
Agitation
Impaired consciousness
Seizures and status epilepticus
Central apnea
Overstimulation of nicotinic receptors in the neuromuscular junction
Muscular weakness
Paralysis of the respiratory muscles
Fasciculations

## Data Availability

No new data were created or analyzed in this study.

## References

[1] Mdeni N.L., Adeniji A.O., Okoh A.I., Okoh O.O. (2022). Analytical Evaluation of Carbamate and Organophosphate Pesticides in Human and Environmental Matrices: A Review. Molecules.

[2] Newmark C.J. (2004). The birth of nerve agent warfare. Neurology.

[3] Eddleston M., Buckley N.A., Eyer P., Dawson A.H. (2008). Management of acute organophosphorus pesticide poisoning. Lancet.

[4] King A.M., Aaron C.K. (2015). Organophosphate and carbamate poisoning. Emerg. Med. Clin. N. Am..

[5] Newmark J. (2004). Therapy for nerve agent poisoning. Arch. Neurol..

[6] Niquet J., Nguyen D., de Araujo Furtado M., Lumley L. (2023). Treatment of cholinergic-induced status epilepticus with polytherapy targeting GABA and glutamate receptors. Epilepsia Open.

[7] Newmark J. (2019). Therapy for acute nerve agent poisoning: An update. Neurol. Clin. Pract..

[8] Gunnell D., Eddleston M., Phillips M.R., Konradsen F. (2007). The global distribution of fatal pesticide self-poisoning: Systematic review. BMC Public Health.

[9] Figueiredo T.H., Apland J.P., Braga M.F.M., Marini A.M. (2018). Acute and long-term consequences of exposure to organophosphate nerve agents in humans. Epilepsia.

[10] Meyer C., Thippeswamy T. (2025). Organophosphate Chemical Nerve Agents, Oxidative Stress, and NADPH Oxidase Inhibitors: An Overview. Int. J. Mol. Sci..

[11] Peter J.V., Sudarsan T.I., Moran J.L. (2014). Clinical features of organophosphate poisoning: A review of different classification systems and approaches. Indian J. Crit. Care Med..

[12] Munidasa U.A., Gawarammana I.B., Kularatne S.A., Kumarasiri P.V., Goonasekera C.D. (2004). Survival pattern in patients with acute organophosphate poisoning receiving intensive care. J. Toxicol. Clin. Toxicol..

[13] Eyer P. (2003). The Role of Oximes in the Management of Organophosphorus Pesticide Poisoning. Toxicol. Rev..

[14] de Araujo Furtado M., Rossetti F., Chanda S., Yourick D. (2012). Exposure to nerve agents: From status epilepticus to neuroinflammation, brain damage, neurogenesis and epilepsy. Neurotoxicology.

[15] McDonough J.H., Shih T.M. (1997). Neuropharmacological mechanisms of nerve agent-induced seizure and neuropathology. Neurosci. Biobehav. Rev..

[16] Gage M., Rao N.S., Samidurai M., Putra M., Vasanthi S.S., Meyer C., Wang C., Thippeswamy T. (2021). Soman (GD) Rat Model to Mimic Civilian Exposure to Nerve Agent: Mortality, Video-EEG Based Status Epilepticus Severity, Sex Differences, Spontaneously Recurring Seizures, and Brain Pathology. Front. Cell. Neurosci..

[17] Blair R.E., Hawkins E., Pinchbeck L.R., DeLorenzo R.J., Deshpande L.S. (2024). Chronic Epilepsy and Mossy Fiber Sprouting Following Organophosphate-Induced Status Epilepticus in Rats. J. Pharmacol. Exp. Ther..

[18] Niquet J., Baldwin R., Suchomelova L., Lumley L., Eavey R., Wasterlain C.G. (2017). Treatment of experimental status epilepticus with synergistic drug combinations. Epilepsia.

[19] Wasterlain C.G., Baldwin R., Naylor D.E., Thompson K.W., Suchomelova L., Niquet J. (2011). Rational polytherapy in the treatment of acute seizures and status epilepticus. Epilepsia.

[20] Burman R.J., Rosch R.E., Wilmshurst J.M., Sen A., Ramantani G., Akerman C.J., Raimondo J.V. (2022). Why won’t it stop? The dynamics of benzodiazepine resistance in status epilepticus. Nat. Rev. Neurol..

[21] Naylor D.E. (2023). In the fast lane: Receptor trafficking during status epilepticus. Epilepsia Open.

[22] Niquet J., Lumley L., Baldwin R., Rossetti F., Schultz M., de Araujo Furtado M., Suchomelova L., Naylor D., Franco-Estrada I., Wasterlain C.G. (2019). Early polytherapy for benzodiazepine-refractory status epilepticus. Epilepsy Behav..

[23] Naylor D.E., Liu H., Wasterlain C.G. (2005). Trafficking of GABA(A) receptors, loss of inhibition, and a mechanism for pharmacoresistance in status epilepticus. J. Neurosci..

[24] Marrero-Rosado B.M., de Araujo Furtado M., Kundrick E.R., Walker K.A., Stone M.F., Schultz C.R., Nguyen D.A., Lumley L.A. (2020). Ketamine as adjunct to midazolam treatment following soman-induced status epilepticus reduces seizure severity, epileptogenesis, and brain pathology in plasma carboxylesterase knockout mice. Epilepsy Behav..

[25] Reddy D.S., Zaayman M., Kuruba R., Wu X. (2021). Comparative profile of refractory status epilepticus models following exposure of cholinergic agents pilocarpine, DFP, and soman. Neuropharmacology.

[26] Williamson J., Singh T., Kapur J. (2019). Neurobiology of organophosphate-induced seizures. Epilepsy Behav..

[27] Reddy D.S. (2024). Neurosteroids as Novel Anticonvulsants for Refractory Status Epilepticus and Medical Countermeasures for Nerve Agents: A 15-Year Journey to Bring Ganaxolone from Bench to Clinic. J. Pharmacol. Exp. Ther..

[28] Kokate T.G., Cohen A.L., Karp E., Rogawski M.A. (1996). Neuroactive steroids protect against pilocarpine- and kainic acid-induced limbic seizures and status epilepticus in mice. Neuropharmacology.

[29] Meletti S., Lucchi C., Monti G., Giovannini G., Bedin R., Trenti T., Rustichelli C., Biagini G. (2017). Decreased allopregnanolone levels in cerebrospinal fluid obtained during status epilepticus. Epilepsia.

[30] Costa A.M., Lucchi C., Simonini C., Lustosa Í.R., Biagini G. (2020). Status Epilepticus Dynamics Predicts Latency to Spontaneous Seizures in the Kainic Acid Model. Cell. Physiol. Biochem..

[31] MacMahon J.A., Andrew P.M., Izadi A., Bruun D.A., Saito N.H., Tancredi D.J., Brooks-Kayal A., Lein P.J., Gurkoff G.G. (2025). Acute and persistent changes in neural oscillatory activity predict development of epilepsy following acute organophosphate intoxication in adult rats. Epilepsia.

[32] Garduño J., Galindo-Charles L., Jiménez-Rodríguez J., Galarraga E., Tapia D., Mihailescu S., Hernandez-Lopez S. (2012). Presynaptic α4β2 nicotinic acetylcholine receptors increase glutamate release and serotonin neuron excitability in the dorsal raphe nucleus. J. Neurosci..

[33] Chen M.W., Zhu H., Xiong C.H., Li J.B., Zhao L.X., Chen H.Z., Qiu Y. (2020). PKC and Ras are Involved in M1 Muscarinic Receptor-Mediated Modulation of AMPA Receptor GluA1 Subunit. Cell. Mol. Neurobiol..

[34] Nowak L., Bregestovski P., Ascher P., Herbet A., Prochiantz A. (1984). Magnesium gates glutamate-activated channels in mouse central neurones. Nature.

[35] Pulkrabkova L., Svobodova B., Konecny J., Kobrlova T., Muckova L., Janousek J., Pejchal J., Korabecny J., Soukup O. (2023). Neurotoxicity evoked by organophosphates and available countermeasures. Arch. Toxicol..

[36] Magro G., Laterza V. (2024). Status epilepticus: Is there a Stage 1 plus?. Epilepsia.

[37] Magro G. (2025). Early Polytherapy for Probably Benzodiazepine Refractory Naïve Status Epilepticus (Stage 1 Plus). Neurol. Int..

[38] Magro G. (2026). The Stage 1 Plus hypothesis in status epilepticus: Predicting resistance before it happens. Med. Hypotheses.

[39] Magro G. (2026). Stage 1 Plus: Toward a unified operational framework in Status Epilepticus. Epilepsia.

[40] Jindal M., Neligan A., Rajakulendran S. (2023). Early and established status epilepticus: The impact of timing of intervention, treatment escalation and dosing on outcome. Seizure.

[41] Llauradó A., Quintana M., Ballvé A., Campos D., Fonseca E., Abraira L., Toledo M., Santamarina E. (2021). Factors associated with resistance to benzodiazepines in status epilepticus. J. Neurol. Sci..

[42] Wasterlain C.G., Niquet J., Lumley L. (2025). Status epilepticus: Is there a stage 1 plus?. Epilepsia.

[43] Rosman Y., Eisenkraft A., Milk N., Shiyovich A., Ophir N., Shrot S., Kreiss Y., Kassirer M. (2014). Lessons learned from the Syrian sarin attack: Evaluation of a clinical syndrome through social media. Ann. Intern. Med..

[44] Baker D.J. (2005). Critical care requirements after mass toxic agent release. Crit. Care Med..

[45] Okudera H. (2002). Clinical features on nerve gas terrorism in Matsumoto. J. Clin. Neurosci..

[46] Okumura T., Takasu N., Ishimatsu S., Miyanoki S., Mitsuhashi A., Kumada K., Tanaka K., Hinohara S. (1996). Report on 640 victims of the Tokyo subway sarin attack. Ann. Emerg. Med..

[47] Okumura T., Suzuki K., Fukuda A., Kohama A., Takasu N., Ishimatsu S., Hinohara S. (1998). The Tokyo subway sarin attack: Disaster management, Part 1: Community emergency response. Acad. Emerg. Med..

[48] Gunderson C.H., Lehmann C.R., Sidell F.R., Jabbari B. (1992). Nerve agents. Neurology.

[49] Moshiri M., Darchini-Maragheh E., Balali-Mood M. (2012). Advances in toxicology and medical treatment of chemical warfare nerve agents. DARU J. Pharm. Sci..

[50] Sekijima Y., Morita H., Yanagisawa N. (1997). Follow-up of sarin poisoning in Matsumoto. Ann. Intern. Med..

[51] Mummadi M.K., Pandurangi R., Geddam J.J.B., Sinha S.N., Rajendran A., Sivaperumal P., Ramachandrappa N.K., Sree Ramakrishna K., Sreenu P. (2021). Investigation of an acute neurological outbreak in Eluru, India, 2020. PLoS ONE.

[52] Exner C.J., Ayala G.U. (2009). Organophosphate and carbamate intoxication in La Paz, Bolivia. J. Emerg. Med..

[53] Wu Y.J., Chang S.S., Chen H.Y., Tsai K.F., Lee W.C., Wang I.K., Lee C.H., Chen C.Y., Liu S.H., Weng C.H. (2023). Human Poisoning with Chlorpyrifos and Cypermethrin Pesticide Mixture: Assessment of Clinical Outcome of Cases Admitted in a Tertiary Care Hospital in Taiwan. Int. J. Gen. Med..

[54] Liu H.-F., Ku C.-H., Chang S.-S., Chang C.-M., Wang I.-K., Yang H.-Y., Weng C.-H., Huang W.-H., Hsu C.-W., Yen T.-H. (2020). Outcome of patients with chlorpyrifos intoxication. Hum. Exp. Toxicol..

[55] Yu J.R., Hou Y.C., Fu J.F., Wang I.K., Chan M.J., Chen C.Y., Weng C.H., Huang W.H., Yang H.Y., Hsu C.W. (2021). Outcomes of elderly patients with organophosphate intoxication. Sci. Rep..

[56] Chuang C.S., Yang K.W., Yen C.M., Lin C.L., Kao C.H. (2019). Risk of Seizures in Patients with Organophosphate Poisoning: A Nationwide Population-Based Study. Int. J. Environ. Res. Public Health.

[57] Eddleston M., Eyer P., Worek F., Mohamed F., Senarathna L., von Meyer L., Juszczak E., Hittarage A., Azhar S., Dissanayake W. (2005). Differences between organophosphorus insecticides in human self-poisoning: A prospective cohort study. Lancet.

[58] Davies J., Roberts D., Eyer P., Buckley N., Eddleston M. (2008). Hypotension in severe dimethoate self-poisoning. Clin. Toxicol..

[59] Eddleston M., Worek F., Eyer P., Thiermann H., Von Meyer L., Jeganathan K., Sheriff M.H., Dawson A.H., Buckley N.A. (2009). Poisoning with the S-Alkyl organophosphorus insecticides profenofos and prothiofos. QJM Int. J. Med..

[60] Vimalanathan S., Ruwanpathirana P., Chang T. (2025). Delayed-onset status epilepticus without cholinergic features in organophosphate poisoning: A case report. Toxicol. Rep..

[61] Zoofaghari S., Maghami-Mehr A., Abdolrazaghnejad A. (2024). Organophosphate Poisoning: Review of Prognosis and Management. Adv. Biomed. Res..

[62] Connors N.J., Harnett Z.H., Hoffman R.S. (2014). Comparison of current recommended regimens of atropinization in organophosphate poisoning. J. Med. Toxicol..

[63] Wang L., Wang X. (2025). Emergency adjunctive therapy for organophosphate poisoning: A meta-analysis. Int. Emerg. Nurs..

[64] Patel A., Chavan G., Nagpal A.K. (2024). Navigating the Neurological Abyss: A Comprehensive Review of Organophosphate Poisoning Complications. Cureus.

[65] Chen X.P., Chen W.Z., Wang F.S., Liu J.X. (2012). Selective cognitive impairments are related to selective hippocampus and prefrontal cortex deficits after prenatal chlorpyrifos exposure. Brain Res..

[66] Bernardino P.N., Luo A.S., Andrew P.M., Unkel C.M., Gonzalez M.I., Gelli A., Lein P.J. (2024). Evidence Implicating Blood-Brain Barrier Impairment in the Pathogenesis of Acquired Epilepsy following Acute Organophosphate Intoxication. J. Pharmacol. Exp. Ther..

[67] Nguyen K.M.C., Swami D., Goyal N., Jalwadi M.G., Acharya M.M., Baulch J.E. (2025). Neurocognitive and neurobiological effects of low dose organophosphate exposure. Neurotherapeutics.

[68] Chen Y.C., Lin C.H., Wu S.L. (2021). Neurological Sequela of Acute Pesticide Poisoning Among Adults in Central Taiwan. Front. Neurol..

[69] Ramírez-Santana M., Zúñiga-Venegas L., Corral S., Roeleveld N., Groenewoud H., Van der Velden K., Scheepers P.T.J., Pancetti F. (2020). Reduced neurobehavioral functioning in agricultural workers and rural inhabitants exposed to pesticides in northern Chile and its association with blood biomarkers inhibition. Environ. Health.

[70] Gao Y., Li R., Ma Q., Baker J.M., Rauch S., Gunier R.B., Mora A.M., Kogut K., Bradman A., Eskenazi B. (2024). Childhood exposure to organophosphate pesticides: Functional connectivity and working memory in adolescents. Neurotoxicology.

[71] Sagiv S.K., Baker J.M., Rauch S., Gao Y., Gunier R.B., Mora A.M., Kogut K., Bradman A., Eskenazi B., Reiss A.L. (2024). Prenatal and childhood exposure to organophosphate pesticides and functional brain imaging in young adults. Environ. Res..

[72] Quintana-Mejia M., Palacio-Herrera F., Olivero-Verbel J., Caballero-Gallardo K. (2025). Exposure to pesticides and cognitive function in school-age children of the Bolivar department (Colombia). Toxicol. Lett..

[73] Fu D., Liu X., Kang N., Cheng X., Chang X., Guo S., Jin H., Zhang Q., Tian Y., Zhang J. (2026). Impact of prenatal exposure to organophosphate flame retardants and organophosphorus pesticides on neurodevelopment in four-year-old children: Evidence from the Shanghai birth cohort. Environ. Int..

[74] Sarailoo M., Afshari S., Asghariazar V., Niri M.V., Safarzadeh E., Dadkhah M. (2025). Diazinon-induced Cognitive Impairment: Alternations in Amyloid Precursor Proteins, and TNF-α Expression in the Hippocampus of Adult Rats. Curr. Top. Med. Chem..

[75] Terry A.V., Beck W.D., Zona V., Itokazu Y., Tripathi A., Madeshiya A.K., Pillai A. (2024). Acute exposure to diisopropylfluorophosphate in mice results in persistent cognitive deficits and alterations in senescence markers in the brain. Front. Neurosci..

[76] Andrew P.M., Lein P.J. (2021). Neuroinflammation as a Therapeutic Target for Mitigating the Long-Term Consequences of Acute Organophosphate Intoxication. Front. Pharmacol..

[77] Cui J., Xiao S., Cao Y., Zhang Y., Yang J., Zheng L., Zhao F., Liu X., Liu D., Zhou Z. (2024). Organophosphate Insecticide Malathion Induces Alzheimer’s Disease-Like Cognitive Impairment in Mice: Evidence of the Microbiota-Gut-Brain Axis. Environ. Sci. Technol..

[78] Flannery B.M., Bruun D.A., Rowland D.J., Banks C.N., Austin A.T., Kukis D.L., Li Y., Ford B.D., Tancredi D.J., Silverman J.L. (2016). Persistent neuroinflammation and cognitive impairment in a rat model of acute diisopropylfluorophosphate intoxication. J. Neuroinflamm..

[79] Guignet M., Dhakal K., Flannery B.M., Hobson B.A., Zolkowska D., Dhir A., Bruun D.A., Li S., Wahab A., Harvey D.J. (2020). Persistent behavior deficits, neuroinflammation, and oxidative stress in a rat model of acute organophosphate intoxication. Neurobiol. Dis..

[80] Sangkarit N., Thammachai A., Suwannakul B., Hongsibsong S., Rohitrattana J., Panumasvivat J., Assavanopakun P., Sapbamrer R. (2025). Neurobehavioral performance of dual exposure to organophosphate pesticides and PAHs among farmers in rural agriculture communities. J. Expo. Sci. Environ. Epidemiol..

[81] Massey N., Vasanthi S.S., Meyer C., Rao N.S., Thedens D.R., Wang C., Kannurpatti S., Thippeswamy T. (2026). Dual targeting of iNOS and Src tyrosine kinase as a superior therapeutic strategy against soman-induced long-term neurotoxicity: Multimodal biomarker, imaging, and neurobehavioral outcome analyses. Acta Neuropathol. Commun..

[82] Ramakrishnan S., Singh T., Reddy D.S. (2024). Protective Activity of Novel Hydrophilic Synthetic Neurosteroids on Organophosphate Status Epilepticus-induced Chronic Epileptic Seizures, Non-Convulsive Discharges, High-Frequency Oscillations, and Electrographic Ictal Biomarkers. J. Pharmacol. Exp. Ther..

